# Supplementary material for: Maternal Trypanosoma cruzi infection is associated with significant placental remodeling regardless of vertical transmission
Source: bioRxiv. 2026 Jan 13:2026.01.13.699142. Preprint. [Version 1] doi: 10.64898/2026.01.13.699142 (PMC12871174; doi:10.64898/2026.01.13.699142)
Supplement: Supplement 1 [file NIHPP2026.01.13.699142v1-supplement-1.pdf]

Supplementary information:

Supplementary Figures:

Supplementary Figure 1. Spatial cell type abundance.
Supplementary Figure 2. Cell type abundance ratio.
Supplementary Figure 3. Cell-to-cell colocalization based on the Jaccard index**.**
Supplementary Figure 4. UMAP plot summarizing gene expression variance.

Supplementary Files:

Supplementary File 1: Differential Gene Expression Analysis DESeq2 results
Supplementary File 2: GSEA by molecular function and biological processes
Supplementary File 3: Manually annotated genes for DEG detected in the placental transcriptome of transmitter mothers.
Supplementary File 4: Metascape analysis of cytotrophoblast and secondary villi enriched regions for spatial transcriptome.
Supplementary File 5: Correlation of blood and placental gene expression transmitter mothers.

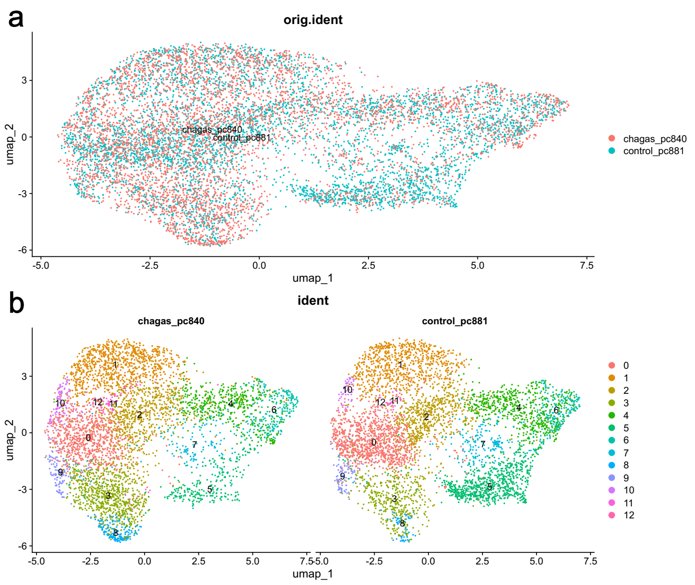

Supplementary Figure 1. UMAP plot summarizing gene expression variance. **a)** gene variance after sample-to-sample normalization using function FindNeighbors function. **b)** gene expression clusters identified using function FindClusters. Analysis was performed using the Seraut package in R.

bioRxiv preprint doi: https://doi.org/10.64898/2026.01.13.699142; this version posted January 13, 2026. The copyright holder has placed this preprint (which was not certified by peer review) in the Public Domain. It is no longer restricted by copyright. Anyone can legally share, reuse, remix, or adapt this material for any purpose without crediting the original authors.

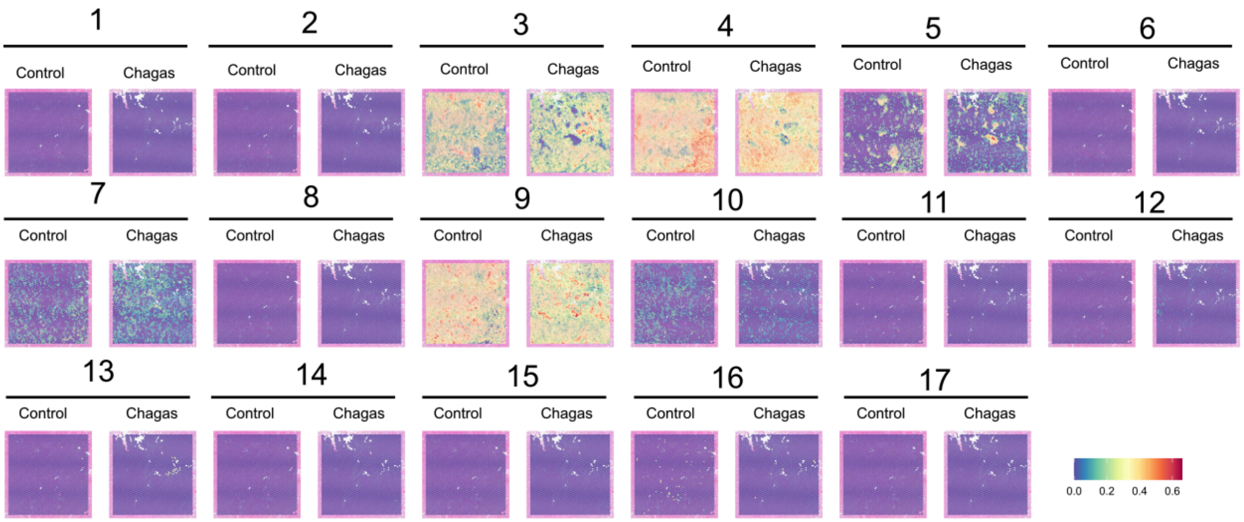

Supplementary Figure 2. Spatial cell type enrichment per sample. Weights of a cell type in a given spot are represented using a universal scale: low abundance in blue, high abundance in red. Cell types: (1) Fetal CD14+ Monocytes, (2) Fetal CD8+ Cytotoxic T Cells, (3) Fetal Cytotrophoblasts, (4) Fetal Endothelial Cells, (5) Fetal Fibroblasts, (6) Fetal GZMB+ Natural Killer, (7) Fetal Hofbauer Cells, (8) Fetal Memory CD4+ T Cells, (9) Fetal Mesenchymal Stem Cells, (10) Fetal Nucleated Red Blood Cells, (11) Fetal Plasmacytoid Dendritic Cells, (12) Fetal Proliferative Cytotrophoblasts, (13) Fetal Syncytiotrophoblast, (14) Maternal CD14+ Monocytes, (15) Maternal CD8+ Cytotoxic T Cells, (16) Maternal FCGR3A+ Monocytes, (17) Maternal Naive CD4+ T Cells.

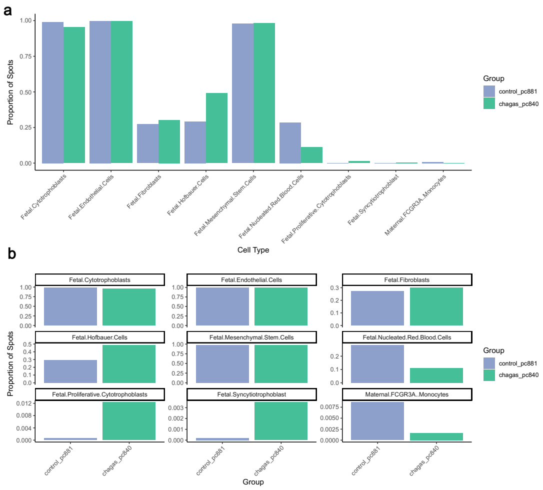

Supplementary Figure 3. Cell type abundance per sample. A. Bar plot summarizing cell type abundance B. Facet plot summarizing cell type abundance with an independent scale per cell type. Only the nine most abundant cell populations are displayed.

bioRxiv preprint doi: https://doi.org/10.64898/2026.01.13.699142; this version posted January 13, 2026. The copyright holder has placed this preprint (which was not certified by peer review) in the Public Domain. It is no longer restricted by copyright. Anyone can legally share, reuse, remix, or adapt this material for any purpose without crediting the original authors.

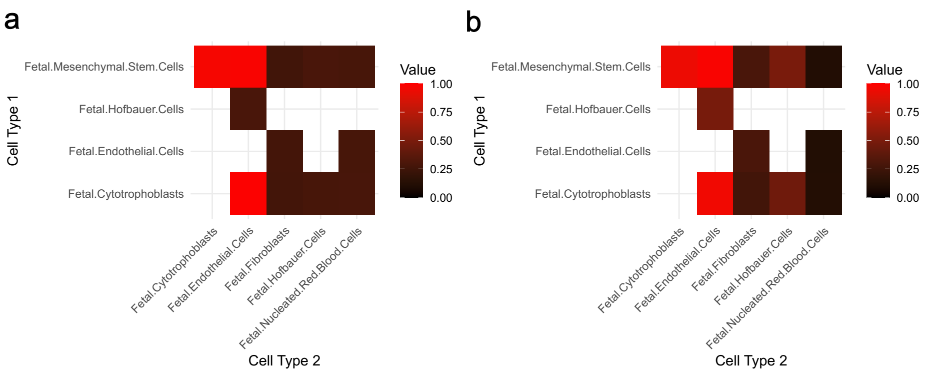

Supplementary Figure 4. Cell-to-cell colocalization based on Jaccard index**. A.** placenta tissue from uninfected control. **B.** placenta tissue from Chagas patient. Only cells exhibiting at least one colocalization with another cell are displayed.

Manual classification of placental DEGs from congenital transmitters into four major biological processes based on bulk RNA-seq.

Category	Gene	Fold change	Description	Role
Extracellular matrix composition/interaction	FILIP1L	**Up (6.49)**	filamin A interacting protein 1 like	Involved in cytoskeleton organization and may regulate cell motility.
	PLOD2	**Up (5.37)**	procollagen-lysine,2-oxoglutarate 5-dioxygenase 2	Plays a role in collagen biosynthesis and cross-linking in the extracellular matrix.
	MGP	**Up (8.65)**	matrix Gla protein	Regulates calcification in the extracellular matrix.
	FAP	**Up (9.15)**	fibroblast activation protein alpha	Associated with extracellular matrix remodeling and fibrosis.
	LUM	**Up (15.28**	lumican	Affects collagen fibril organization in the extracellular matrix.
	FLRT2	**Up (4.37)**	fibronectin leucine rich transmembrane protein 2	Regulates cell adhesion and interactions with the extracellular matrix.
	DMD	**Up (3.12)**	Dystrophin	Maintains the structural integrity of muscle fibers by connecting the cytoskeleton to the extracellular matrix.
	LAMA5	**Down (-2.97)**	Laminin Subunit Alpha 5	Integral to basement membrane structure and function, contributing to cell adhesion, migration, and differentiation within the extracellular matrix.
	FGFR3	**Down (-3.29)**	Fibroblast Growth Factor Receptor 3	A key regulator in cell growth, differentiation, and repair through fibroblast growth factor signaling.
Cell proliferation modulators	MAPK1	**Up (4.12)**	mitogen-activated protein kinase 1	Plays a critical role in the MAPK/ERK signaling pathway, which is directly involved in cell growth and proliferation.
	FGFR3	**Down (-3.29)**	fibroblast growth factor receptor 3	A fibroblast growth factor receptor involved in regulating cell proliferation, differentiation, and apoptosis.
	ANGPTL1		angiopoietin like 1	Participate in the formation of blood vessel.
	STAT1	**Up (4.06)**	signal transducer and activator of transcription 1	A transcription factor involved in cellular responses to growth factors and cytokines, affecting proliferation.
	NFKB1	**Up (2.54)**	nuclear factor kappa B subunit 1	Central in the NF-κB signaling pathway, which regulates cell proliferation, survival, and immune response.
	RPS27A, RPL9, RPL21, RPL35A, and RPS3A	**RPS27A (3.38), RPL9 (4.75)**, **RPL21 (3.54)**, **RPL35A (4.41)**, and **RPS3A(4.05)**		Ribosomal proteins essential for protein synthesis, indirectly contributing to cell proliferation.
	EIF2A	**Up (6.14)**	eukaryotic translation initiation factor 2A	Plays a role in translation initiation, critical for cell growth and proliferation.
	USP16	**Up (5.50)**	ubiquitin specific peptidase 16	Regulates histone H2A deubiquitination, important for cell cycle progression and proliferation.
	MED21	**Up (4.25)**	mediator complex subunit 21	Part of the Mediator complex, which regulates transcription of genes involved in cell proliferation.
	CAPZA1	**Up (9.04)**	capping actin protein of muscle Z-line subunit alpha 1	A capping protein involved in actin filament dynamics, important for cell division and proliferation.
	CAND1	**Up (3.45)**	cullin associated and	Regulates the ubiquitin-proteasome system, influencing cell cycle and proliferation.

bioRxiv preprint doi: https://doi.org/10.64898/2026.01.13.699142; this version posted January 13, 2026. The copyright holder has placed this preprint (which was not certified by peer review) in the Public Domain. It is no longer restricted by copyright. Anyone can legally share, reuse, remix, or adapt this material for any purpose without crediting the original authors.

			neddylation dissociated 1	
	TMEM62	**Up (3.6)**	transmembrane protein 62	Associated with cell cycle and growth processes.
	DPP4	**Up (4.34)**	dipeptidyl peptidase 4	Known to regulate proliferation in certain cell types through signaling pathways.
	FOXP1	**Up (2.72)**	forkhead box P1	A transcription factor linked to cell cycle regulation and proliferation.
	MAP3K2	**Up (3.41)**	mitogen-activated protein kinase kinase kinase 2	Involved in MAP kinase signaling pathways that regulate cell proliferation and survival.
	COPS4, COPS8	**Up(11.18) Up(3.43)**		Components of the COP9 signalosome, which influences cell cycle and proliferation
Inflammation	NFKB1	**Up (2.54)**	Nuclear Factor Kappa B Subunit 1	A master regulator of proinflammatory gene expression.
	ERAP2	**Up (6.60)**	endoplasmic reticulum aminopeptidase 2	Involved in antigen processing and presentation, which is crucial for activating immune responses, including inflammation
	B2M	**Up (9.53)**	Beta-2-Microglobulin	Plays a role in the MHC class I antigen presentation pathway, directly influencing immune activation.
	TLR1	**Up (22.6)**	Toll-Like Receptor 1	Recognizes pathogen-associated molecular patterns (PAMPs) and activates proinflammatory signaling cascades.
	TLR7	**Up (10.53)**	Toll-Like Receptor 7	TLR7 is a critical innate immune receptor involved in recognition of parasite RNA, induction of IL-12p40 by dendritic cells, and consequent IFN-γ by T lymphocytes.
	CTSS	**Up (3.63)**	Cathepsin S	A cysteine protease involved in antigen processing and the regulation of inflammatory responses.
	STAT1	**Up (4.06)**	signal transducer and activator of transcription 1	A transcription factor that mediates responses to proinflammatory cytokines such as interferons.
	MAPK1	**Up (4.12)**	Mitogen-Activated Protein Kinase 1	Part of the MAPK signaling pathway, which is critical for proinflammatory cytokine production.
	FAS	**Up (3.97)**	Fas Cell Surface Death Receptor	Mediates apoptosis and inflammatory signaling in immune cells.
	SPP1	**Up (5.17)**	secreted phosphoprotein 1	cytokine that upregulates expression of interferon-gamma and interleukin-12.
	IFI44L	**Up (8.97)**	Interferon Induced Protein 44 Like	Predicted to be involved in immune response.
	NKTR	**Up (2.35)**	Natural Killer Cell Triggering Receptor	It is present on the surface of natural killer cells and facilitates their binding to targets. Its expression is regulated by IL2 activation of the cells.
Apoptosis and DNA damage	MACROD1	**Down (4.65)**	mono-ADP ribosylhydrolase 1	Implicated in the DNA damage response via ADP-ribosylation repair pathways.
	PARP9	**Up (4.43)**	poly(ADP-ribose) polymerase family member 9	Involved in DNA repair and apoptosis regulation.
	CYLD	**Up (2.22)**	CYLD lysine 63 deubiquitinase	A tumor suppressor that regulates apoptosis and DNA damage responses.
	ATR	**Up (5.51)**	ATR serine/threonine kinase	Key player in the DNA damage response, activating cell cycle checkpoints and repair.
	SUMO1	**Up (3.55)**	small ubiquitin like modifier 1	Regulates apoptosis and DNA repair processes through post-translational modifications.
	FAS	**Up (3.97)**	Fas cell surface death receptor	Critical in apoptosis as a death receptor that initiates programmed cell death.

bioRxiv preprint doi: https://doi.org/10.64898/2026.01.13.699142; this version posted January 13, 2026. The copyright holder has placed this preprint (which was not certified by peer review) in the Public Domain. It is no longer restricted by copyright. Anyone can legally share, reuse, remix, or adapt this material for any purpose without crediting the original authors.

	KAT5 (TIP60)	**Down (-2.56)**	lysine acetyltransferase 5	Acetylates proteins involved in the DNA damage response and apoptosis.
	DRAM1	**Up (2.86)**	DNA damage regulated autophagy modulator 1	Mediates autophagy and apoptosis, particularly in response to p53 activation.
	CUL5	**Up (16.17)**	cullin 5	Located in site of DNA damage.
	SOD2	**Up (4.74)**	superoxide dismutase 2	Directly or indirectly in producing DNA damage.

Note: Forty-seven of the 298 unique DEGs were classified into these four major biological processes.

bioRxiv preprint doi: https://doi.org/10.64898/2026.01.13.699142; this version posted January 13, 2026. The copyright holder has placed this preprint (which was not certified by peer review) in the Public Domain. It is no longer restricted by copyright. Anyone can legally share, reuse, remix, or adapt this material for any purpose without crediting the original authors.
